# Epidermolysis bullosa acquisita: an uncommon cause of esophageal stricture

**DOI:** 10.1093/omcr/omab010

**Published:** 2021-04-28

**Authors:** Carlos Figueredo, Konstantin Boroda, Hilary Hertan

**Affiliations:** 1 Department of Internal Medicine, Montefiore Medical Center, Bronx, NY, USA; 2 Division of Gastroenterology and Hepatology, Montefiore Medical Center, Bronx, NY, USA

## Abstract

Epidermolysis bullosa acquisita (EBA) encompasses a wide spectrum of rare diseases with a common genetic origin transmitted in an autosomal recessive fashion. Mild forms of non-inflammatory EBA are characterized by skin lesions and have gained great relevance in the literature. However, resistant inflammatory EBA with widespread mucosal involvement remains a rare entity given its low prevalence. It commonly represents a great burden for the patient’s quality of life with most cases being resistant to different therapeutic modalities. We present a case of resistant inflammatory EBA with esophageal strictures that improved after therapy with intravenous immunoglobulin and rituximab.

## INTRODUCTION

Epidermolysis bullosa acquisita (EBA) is a well-defined acquired autoimmune disease that results from autoantibodies against anchoring fibrils within the basement membrane of collagen type VII [1]. It can be divided into two subtypes: non-inflammatory and inflammatory. Non-inflammatory is the most common subtype, limited to skin lesions in body prone areas. In contrast, the inflammatory subtype has a widespread involvement and affects the mucosal surfaces of the oral cavity, nasopharynx, larynx and esophagus. However, gastrointestinal tract involvement is rare, and its incidence is too low to be determined. We present an uncommon case presentation of EBA with esophageal strictures that improved with dilation, intravenous immunoglobulin (IVIg) and rituximab (RTX) [2, 3].

## CASE REPORT

**Figure 1:**
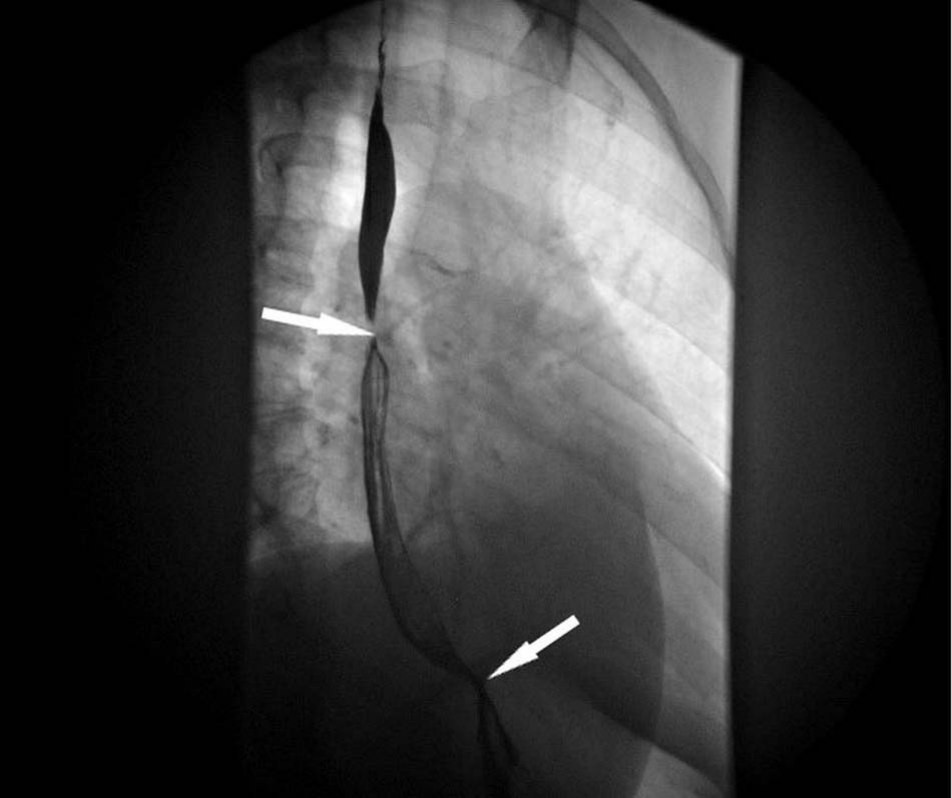
Barium swallow esophagram with multiple areas of stenosis in the upper and lower third of the esophagus.

**Figure 2:**
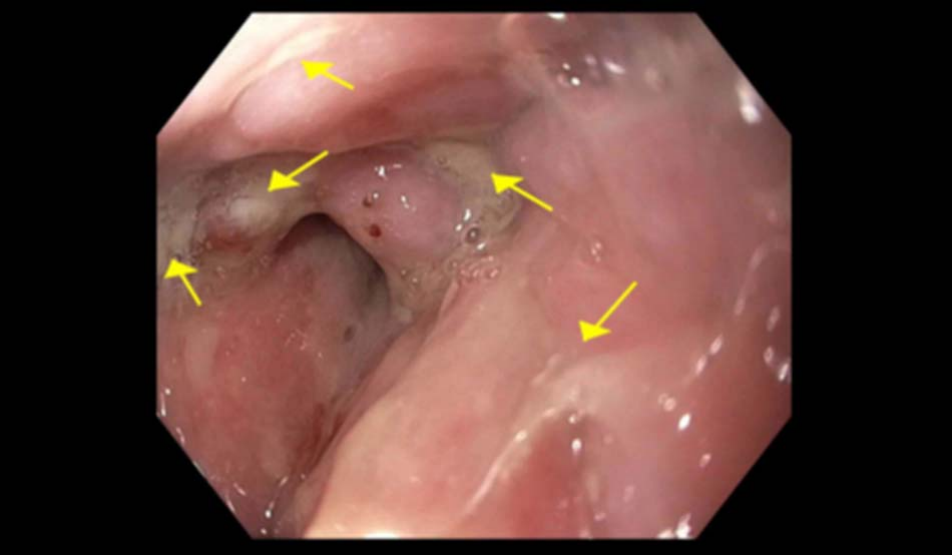
Multiple shallow ulcers in the oropharynx.

**Figure 3:**
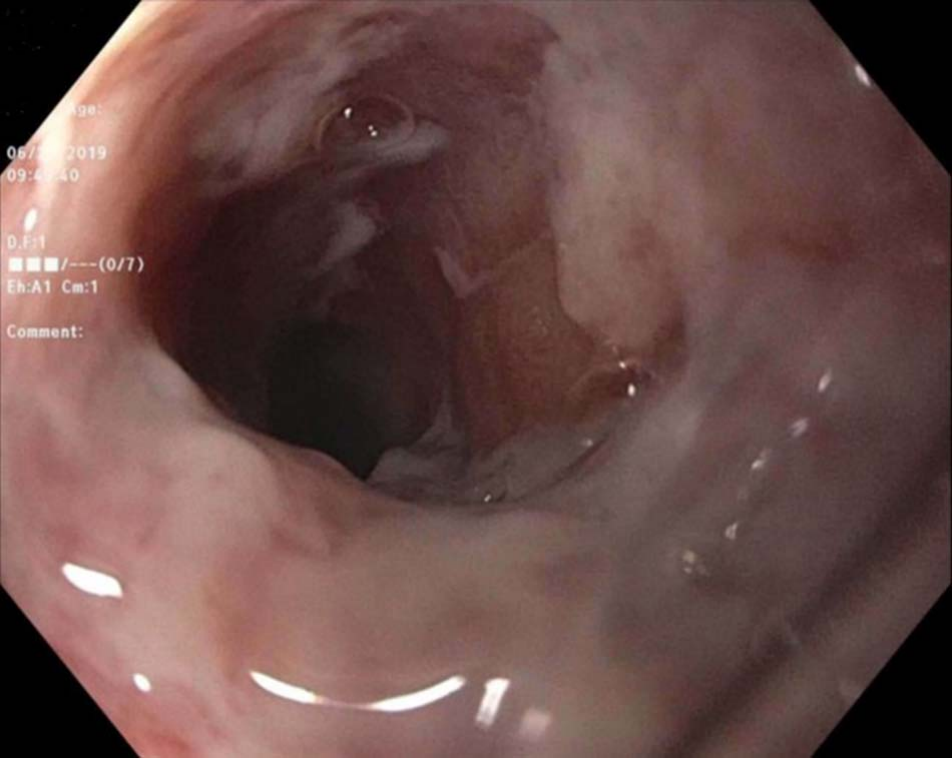
Area of stenosis in the upper third of the esophagus with sloughing of the surrounded mucosa.

**Figure 4:**
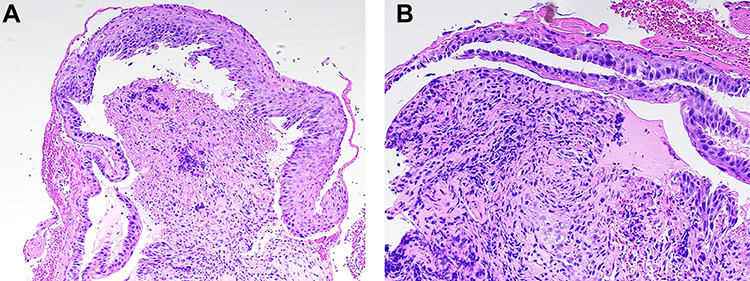
(**a**) Biopsy from the lower third of the esophagus. The area of the epithelium is separated from the underlying stroma. Both the epithelium and the stroma are involved by acute and chronic inflammation not related to infection. (**b**) Biopsy from the lower third esophagus. Fibrin is seen in the space between the epithelium and stroma consistent with non-infectious inflammation and area of stenosis.

A 71-year-old female with EBA and history of Mucosa-associated lymphoid tissue (MALT) lymphoma non-Hodgkin lymphoma of the right orbit in remission presented with non-bilious, non-bloody vomiting; associated dysphagia, odynophagia and unintentional weight loss of 8 lbs in the last 1 month. Physical exam was significant for scattered skin bullae throughout the body. There were oral ulcerations without visible thrush. The patient was started on lidocaine and Maalox cocktail, sucralfate and proton pump inhibitor with partial relief of symptoms. Barium swallow esophagram evidenced multiple stenotic areas (Fig. 1). Esophagogastroduodenoscopy was performed revealing extensive palate and laryngeal ulcers (Fig. 2), along with erosive lesions and esophageal stenosis in the upper and lower third of the esophagus (Fig. 3). The lower esophagus and stomach were spared. The stenotic areas were successfully dilated with Savary dilators. Biopsy revealed ulcerative esophagitis with acute and chronic areas of inflammation that resulted negative for metaplasia, viral and fungal infections (Fig. 4a and b). The patient was initiated on IVIg and continued RTX with symptoms improvement.

Regarding the patient’s EBA, she had a long-standing history of progressive, widespread blisters, along with palate and laryngeal ulcers for the past 3 years, which previously failed steroids and dapsone treatment. Two months before presentation, the patient was started on RTX given the severe, non-remitting course of her EBA. The patient remained in complete remission with no further mucosal involvement for 5 months after the initial presentation and completing 5 cycles of IVIg, and RTX. On follow-up skin biopsy, dermatopathology analysis by immunofluorescence showed no deposition of IgG, IgM, IgA or C3 in the epidermis, dermis, basement membrane zone and vessels of the oral mucosa. The patient was discharged with dermatology and gastroenterology (GI) follow-up. No further GI symptoms were reported after 9 months of follow-up visits.

## DISCUSSION

EBA has a worldwide annual prevalence of 0.2% per 1 million people. It has been previously associated with Crohn’s disease (in 25–30% of reported cases), ulcerative colitis, along with other autoimmune disorders such as B cell lymphoma, amyloidosis, thyroiditis, chronic lymphocytic leukemia and diabetes; out of which, the first three have only been proven by research trials. Histopathological analysis has shown evidence of circulating tissue antibodies against type VII collagen in B cell lymphoma patients [2, 4].

The treatment for EBA depends on the extent of the blistering lesions and their effect on the patient’s quality of life. First-line therapy for mild (local) disease is local vs systemic steroids; or steroid-sparing agents such as colchicine, dapsone, cyclosporine, azathioprine, mycophenolate and cyclophosphamide. The classic feature of inflammatory EBA is the mucosal extension of the blistering lesions, which has been reported in 20% of cases and can be life-threatening. This subtype tends to be refractory to steroids and steroid-sparing agents. Nevertheless, IVIg along with other therapies such as humanized anti CD20 monoclonal antibodies (RTX) have shown positive outcomes [5-7].

Gastrointestinal involvement of EBA is uncommon. The clinical presentation consists of multiple blisters and ulcer formation in the oral mucosa and palate region with progressive dysphagia, and/or odynophagia. There has been an established relationship between esophageal webs and EBA, reported in 6% of cases. The esophageal blistering/scarring process leads to the formation of fibrotic mucosa and esophageal stenosis. Management often encompasses dietary modification, sequential esophageal dilations with a balloon catheter or a bougie, and a multidisciplinary approach, including psychological support. In one study with 53 patients, a total of 182 dilations were performed over a median follow-up period of 3.5 years. In total, 95% of the patients improved in the dysphagia score and weight. Possible risks are esophageal rupture and re-stenosis. Overall, endoscopic dilation is a safe procedure that provides symptom relief and improvement of the quality of life [8].

One meta-analysis designed to study the efficacy of various treatments on remission showed that all EBA cases had a satisfactory response to IVIG and RTX independent of the disease severity. In a subgroup analysis, EBA with mucosal involvement was associated with complete remission. Furthermore, RTX and IVIG exhibited the best profile outcome for complete remission in severe EBA. However, it is not clear which mechanism RTX and IVIg use to execute their effect over susceptible autoantigenic B cell lymphocytes. IVIg is well known for its anti-inflammatory effects, as well as its ability to eliminate clinical autoimmunity and restore physiologic homeostasis. [9]. These two agents might have a synergistic effect, but additional trials with larger samples are required. The length of therapy with IVIg and RTX is still controversial but expert consensus suggests that multiple cycles are necessary with a minimum length of 1 month [10].

The goal of treatment is to control the disease and to avoid new lesion formation. The EBA inflammatory subtype prognosis is poor and 1-year remission off medication has not been widely proven in previous trials. Complete remission is uncommon without at least short courses of medical therapy. Hence, moderate to long-term intensity therapies are needed in most cases to achieve lesser disease activity and remission. Severe forms of EBA have an unfavorable remission profile with frequent exacerbations and rapid scarring, negatively affecting the patient’s quality of life [10].

Our patient had a typical course of inflammatory EBA with GI mucosal involvement. Her condition was refractory to conventional therapy, but later achieved remission for more than 5 months after combination therapy with RTX and IV immunoglobulin.
